# The CPT1a inhibitor, etomoxir induces severe oxidative stress at commonly used concentrations

**DOI:** 10.1038/s41598-018-24676-6

**Published:** 2018-04-19

**Authors:** Roddy S. O’Connor, Lili Guo, Saba Ghassemi, Nathaniel W. Snyder, Andrew J. Worth, Liwei Weng, Yoonseok Kam, Benjamin Philipson, Sophie Trefely, Selene Nunez-Cruz, Ian A. Blair, Carl H. June, Michael C. Milone

**Affiliations:** 1 Center for Cellular Immunotherapies, Perelman School of Medicine at the University of Pennsylvania, Philadelphia, PA USA; 2 Penn SRP center, Center of Excellence in Environmental Toxicology, and Department of Systems Pharmacology and Translational Therapeutics at the University of Pennsylvania, Philadelphia, PA USA; 3 A.J. Drexel Autism Institute, Drexel University, Philadelphia, PA USA; 4 Department of Pathology and Laboratory Medicine, Perelman School of Medicine at the University of Pennsylvania, Philadelphia, PA USA; 5 Agilent Technologies Inc, Lexington, MA USA; 6 University of Pennsylvania School of Medicine, Philadelphia, PA USA

## Abstract

Etomoxir (ETO) is a widely used small-molecule inhibitor of fatty acid oxidation (FAO) through its irreversible inhibitory effects on the carnitine palmitoyl-transferase 1a (CPT1a). We used this compound to evaluate the role of fatty acid oxidation in rapidly proliferating T cells following costimulation through the CD28 receptor. We show that ETO has a moderate effect on T cell proliferation with no observable effect on memory differentiation, but a marked effect on oxidative metabolism. We show that this oxidative metabolism is primarily dependent upon glutamine rather than FAO. Using an shRNA approach to reduce CPT1a in T cells, we further demonstrate that the inhibition of oxidative metabolism in T cells by ETO is independent of its effects on FAO at concentrations exceeding 5 μM. Concentrations of ETO above 5 μM induce acute production of ROS with associated evidence of severe oxidative stress in proliferating T cells. In aggregate, these data indicate that ETO lacks specificity for CTP1a above 5 μM, and caution should be used when employing this compound for studies in cells due to its non-specific effects on oxidative metabolism and cellular redox.

## Introduction

Quiescent T cells undergo major shifts in their metabolism following antigen-induced activation and CD28 costimulation^1^. Increased glucose uptake and a shift towards glycolysis are some of the earliest changes to occur^2^. Although necessary for proliferation, glycolysis also appears to be intimately linked to the differentiation and function of T cells. Enhancing glycolysis in T cells by overexpression of the glycolytic enzyme, phosphoglycerate mutase-1, reduces their ability to form long-lived memory cells^3^. In addition, glyceraldehyde-3-phosphate dehydrogenase (GAPDH), an enzyme central to the glycolytic pathway, plays a secondary role in T cells, regulating the expression of the effector cytokine IFN-γ by binding to the 3′ UTR of nascent cytokine transcripts and suppressing translation when not engaged in glycolysis^4^.

Glutamine and fatty acids are also important nutrients for T cells. Glutamine has long been recognized as a critical amino acid for optimal T cell proliferation *ex vivo*. T cell immunity *in vivo* is also highly dependent upon glutamine transport^5^, and glutamine-derived α-ketoglutarate (aKG) availability influences the differentiation of CD4+ T cells into Th1 vs. regulatory T cells (Tregs)^6^. Fatty acid oxidation (FAO) has also been reported to play important roles in both the development of regulatory T cells and CD8+ memory T cells^7,8^. CD8+ memory T cells have been shown to engage in a futile cycle of fatty acid synthesis and fatty acid oxidation (FAO) that has been postulated to support mitochondrial health and the long-term survival of memory-differentiated cells^9^.

In an effort to explore the role of fatty acid oxidation in the proliferation and differentiation of T cells within a previously well-described *ex vivo* culture system that relies upon CD28 costimulation, we used etomoxir, 2[6(4-chlorophenoxy) hexyl] oxirane-2-carboxylate, an irreversible inhibitor of carnitine palmitoyltransferase 1a (CPT1a). This transporter is critical for the oxidation of long chain fatty acids (LCFA) within mitochondria. Unexpectedly, we observe that ETO has significant off-target effects on T cells at commonly used concentrations including induction of severe oxidative stress. These results have important implications for studies that use ETO as a pharmacologic inhibitor of FAO.

## Results

### Etomoxir reduces CD28-costimulated T cell proliferation without affecting T cell effector differentiation

In general, studies examining the role of LCFAO in T cell memory cell differentiation, as well as macrophage M2 polarization, have used ETO at concentrations ranging from 40 μM–200 μM^8–15^. We began our studies of fatty acid metabolism by using ETO at a 50 μM concentration to inhibit CPT1a-mediated LCFA transport and FAO. This concentration was selected based upon previous studies showing diminished palmitate oxidation in L6 myoblasts and [1-^14^C] palmitate metabolism in H9C2 cells using 50 μM ETO^16,17^.

**Figure 1:**
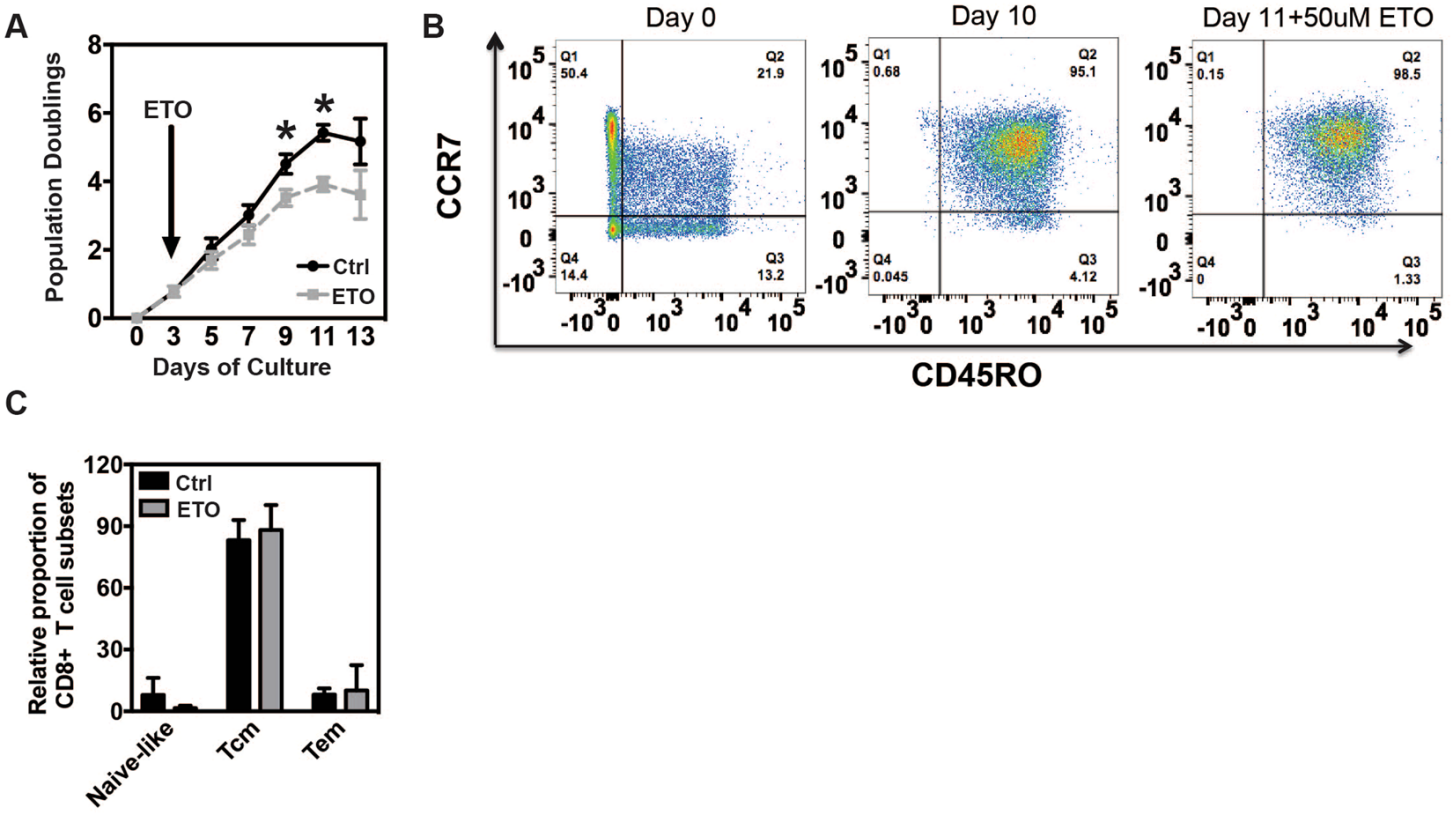
ETO moderately inhibits T cell proliferation with minimal effect on differentiation. (**A**) Activated T cells (day 3) were treated with either control or 50 μM ETO every other day throughout logarithmic expansion. Live cells were enumerated using flow cytometric, bead-based approaches. The number of population doublings is plotted as a function of time. Values are means ± S.E.M. from 7 independent experiments. (*P < 0.05 for control vs ETO). (**B**) Representative plots (n = 6) of cell surface expression of CCR7 and CD45RO on T cells treated with 50 μM ETO for 7 days during logarithmic expansion. Cells shown have been pre-gated for live, CD3+ CD8+ T cells. (**C**) Relative proportion of naïve-like (CCR7+; CD45RO−), Tcm (CCR7+; CD45RO+) and Tem (CCR7−; CD45RO+) subsets (after gating on live, CD3+ CD8+ T cells). Data are plotted as mean ± S.E.M. from 6 separate donors.

We show that primary human T cells cultured *ex vivo* following activation by agonist antibodies to CD3 and CD28 show a modest reduction in the rate of proliferation when cultured in the presence of 50 μM ETO. As seen in Fig. 1A, ETO diminished overall proliferation by approximately 2 fold. Despite their reduced proliferative capacity, control and ETO-treated T cells possessed similar levels of central memory and effector memory surface markers at the end of the *ex vivo* culture (Fig. 1B,C). These results contrast with previously-reported studies in mouse T cells using ETO that have demonstrated a requirement for long-chain FAO in CD8+ memory T cell differentiation^1^.

### Etomoxir inhibits oxidative metabolism of glutamine in CD28-costimulated T cells

**Figure 2:**
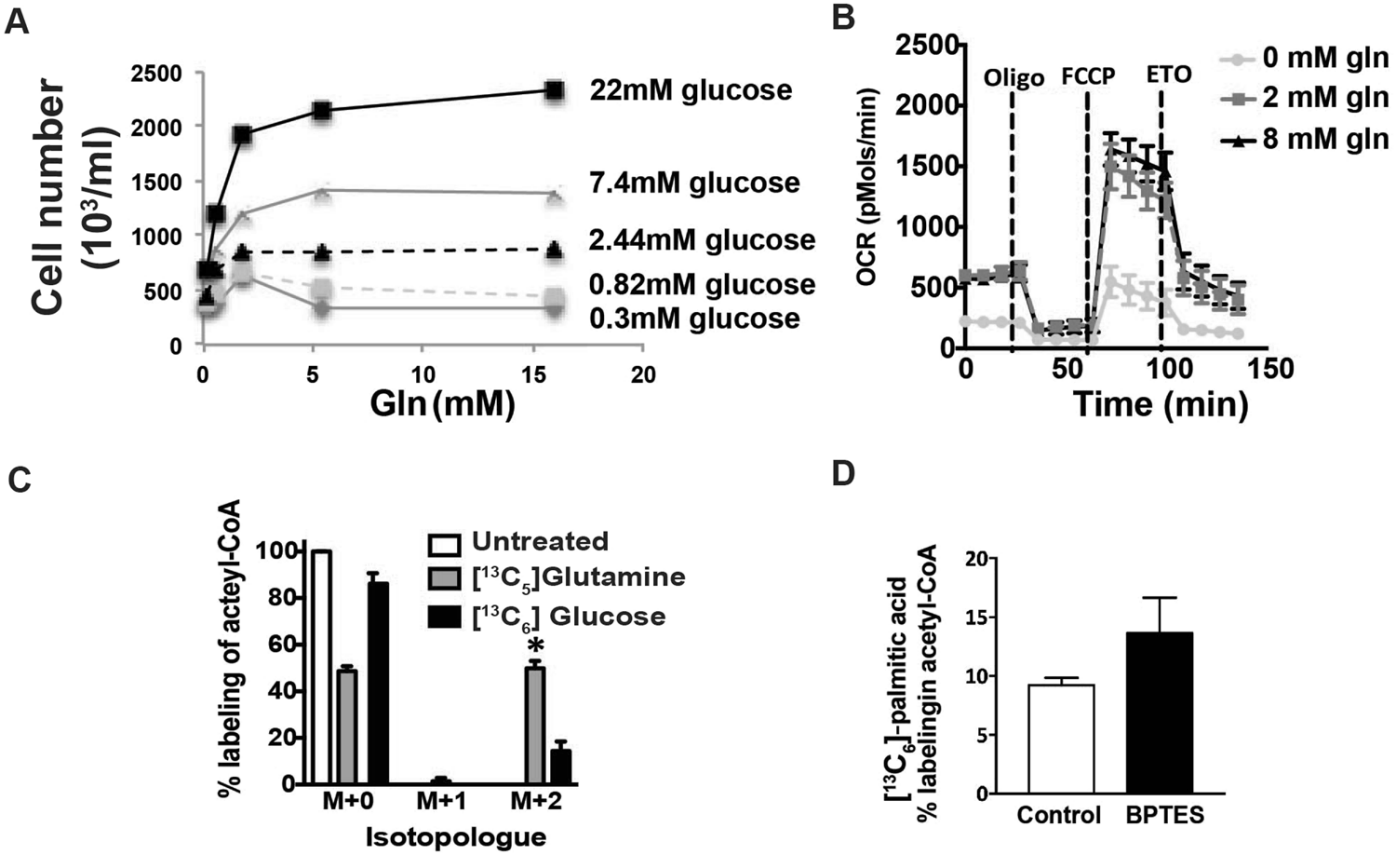
ETO inhibits oxidative glutamine metabolism in CD28-costimulated T cells. (**A**) Activated T cells (day 3) were switched to cell culture medium containing varying levels of glucose and glutamine. After 48 hrs, live cells were enumerated using bead-based flow cytometry, as described in the materials and methods. Representative values from two independent experiments with separate donors are shown. (**B**) T cells were expanded following stimulation with dynabeads. After 7 days, the cells were transferred to bicarbonate-free XF assay medium containing varying concentrations of glutamine. Metabolic parameters were measured by extracellular flux assay (Seahorse). The oxygen consumption rate (OCR) was measured at baseline and following the addition of 1.5 μM oligomycin, 1.5 μM FCCP, and 50 μM ETO. Values are means ± S.E.M. from 3 independent experiments. (**C**) T cells undergoing logarithmic expansion (day 7) were treated with either 10 mM ^13^C_6_ glucose or 2 mM ^13^C_5_ glutamine for 1 hr. Mass isotopomer data for acetyl-CoA are shown. Values represent means ± S.E.M. from 3 independent experiments. (**D**) T cells undergoing logarithmic expansion (day 7) were treated with ^13^C_16_ palmitate +/−10 μM BPTES for 2 hrs. Mass isotopomer data for acetyl-CoA are shown. Values represent means ± S.E.M. from 3 independent experiments.

Based upon the observed effect of ETO on T cell proliferation without an appreciable effect on differentiation, we examined the nutrient requirements of CD28-costimulated primary human T cells further in order to understand the effects of ETO on T cell metabolism. T cells in log-phase proliferation rely upon glucose and glutamine for proliferation (Fig. 2A). Glutamine concentrations of 2 mM are sufficient to support maximal T cell proliferation. In contrast, glucose increased T cell proliferation in a dose-dependent manner. Despite this preferential reliance on glucose for proliferation, mitochondrial oxidative metabolism relies largely upon exogenous glutamine (Fig. 2B). In order to evaluate the contribution of LCFA to T cell oxidative metabolism, we measured oxygen consumption rate (OCR) following the addition of ETO in an extracellular metabolic flux assay. Instead of the typically used mitochondrial electron transport chain complex I inhibitor, rotenone, we used ETO to determine the contribution of LCFA to T cell oxidative metabolism. The OCR of rapidly proliferating T cells is markedly inhibited by ETO, suggesting a high degree of CTP1a-dependent LCFA oxidation (Fig. 2B). The extent to which T cell mitochondrial oxidative metabolism was dependent on LCFA is unexpected given the ability of mammalian cells to generally utilize carbon sources interchangeably. Isotopic labeling studies using ^13^C-labeled glucose and glutamine show that the majority (>50%) of acetyl-CoA and citrate in proliferating T cells is derived from glutamine rather than glucose (Fig. 2C).

Mitochondrial respiration is important for both ATP production as well as biosynthesis within cells by supplying tricarboxylic acid (TCA) cycle intermediates including short chain CoA’s that serve as important precursors for amino acid and fatty acid synthesis^18^. Given the apparent dependency of OCR on both glutamine and fatty acids, we evaluated the contribution of exogenously supplied LCFA to acetyl-CoA pools in rapidly dividing T cells using ^13^C_16_-labeled palmitate. We observed approximately 5% labeling of total acetyl-CoA from the uniformly ^13^C-labeled palmitate (Fig. 2D). Cellular uptake of LCFA did not appear to be limiting FA utilization as the oxidation of exogenous long chain fatty acids doubled when glutamine metabolism was inhibited. In aggregate, these data appeared consistent with the previously reported futile cycle whereby glutamine fuels intracellular fatty acid production, and the newly synthesized fatty acids are transported into the mitochondrial matrix where they undergo beta-oxidation^9^.

### The specificity of etomoxir in T cells is compromised at doses above 5 μM

**Figure 3:**
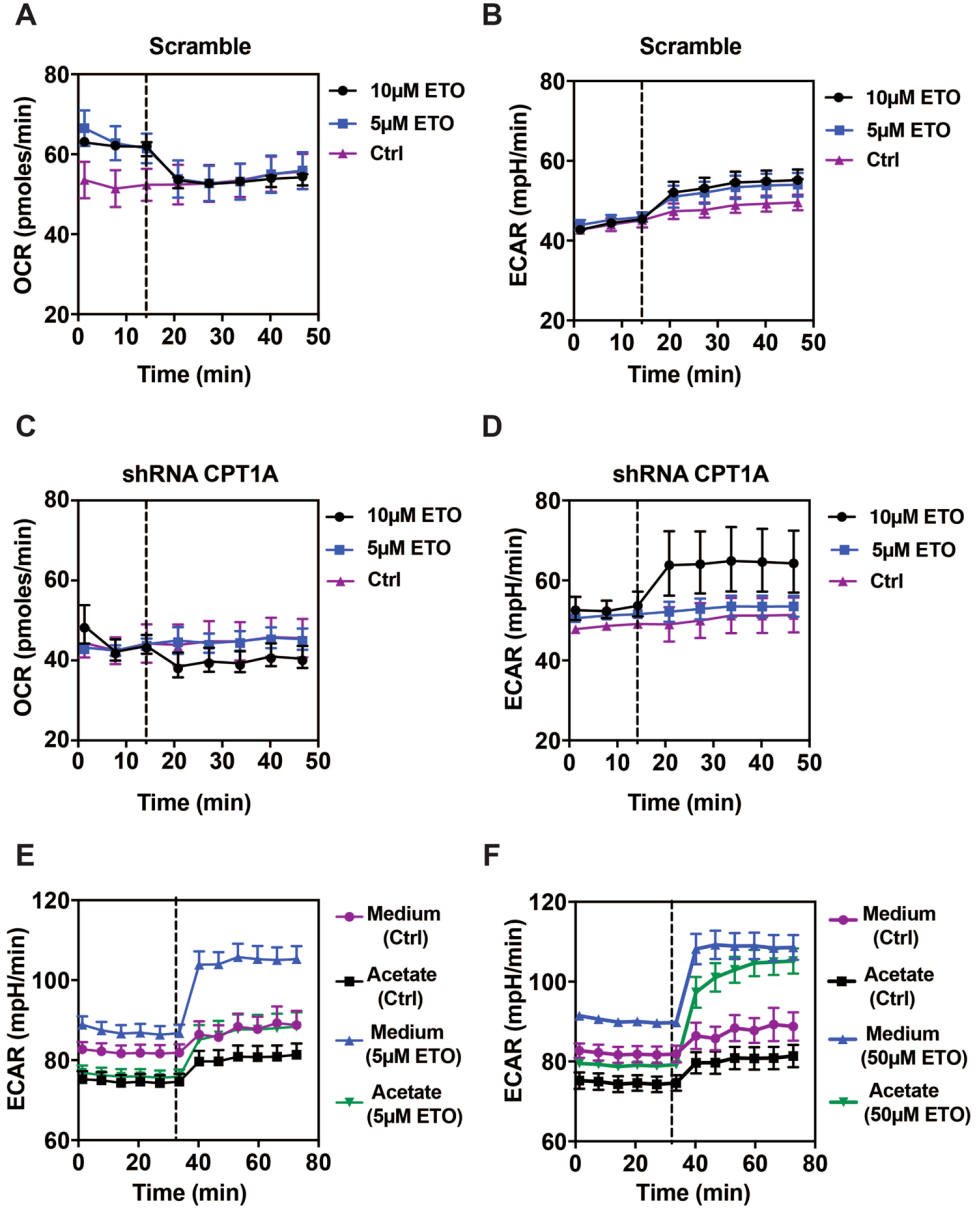
The specificity of ETO for CPT1a is lost at concentrations above 5 μM T cells expressing either control or shRNA against CPT1A were restimulated with dynabeads and expanded for 5 days. Oxygen consumption rates (OCR) in control (**A**) versus shRNA cells (**C**) under basal conditions and after the introduction of ETO (dotted lines) are shown. The corresponding glycolytic rates (ECAR) in control (**B**) and shRNA cells (**D**) cells are shown. Representative metabolic parameters from 2 independent experiments are shown. Untransduced T cells were expanded for 8 days with dynabeads and then switched to regular Seahorse assay medium (Medium) or assay medium supplemented with 1 mM sodium acetate (Acetate). Glycolytic rates were measured under basal conditions and following the introduction of 5 μM ETO (**E**) and 50 μM ETO (**F**) at the indicated time-points (dotted lines). Representative metabolic parameters from 2 independent experiments are shown.

Although glutamine-derived fatty acids could provide an important source of acetyl-CoA for the TCA cycle, concerns about the use of ETO as a “specific” inhibitor of FAO have been raised in the literature^19^. We therefore used a non-pharmacologic approach of shRNA to reduce CPT1a expression and assess the specificity of ETO in primary human T cells. Activated T cells transduced with lentiviral vectors encoding shRNA against CPT1A or a control shRNA (scramble) in combination with GFP were expanded and sorted to >99% purity (data not shown). Similar to ETO, shRNA knockdown also had a small, but measurable effect on T cell proliferative rate and overall expansion (Fig. S1). Concentrations of ETO as low as 5 μM decrease the OCR in control cells expressing the scramble shRNA with a corresponding increase in ECAR (Fig. 3A,B). In the context of cells with reduced CPT1A expression, 5 μM ETO had no effect on either OCR or ECAR (Fig. 3C,D). However, 10 μM ETO diminished the rate of oxygen consumption, and increased ECAR in these cells with reduced CPT1A. These data suggest a CPT1a-independent effect of ETO on oxidative metabolism at concentrations above 5 μM in human T cells. Supporting these studies, we show that when the cell culture medium was supplemented with acetate, a short chain fatty acid whose entry into the mitochondria is not transport-limited, the metabolic effects of 5 μM ETO were reversed (Fig. 3E). However, acetate was unable to reverse the metabolic consequences elicited by higher concentrations of ETO (Fig. 3F).

### Etomoxir promotes oxidative stress in proliferating T cells

**Figure 4:**
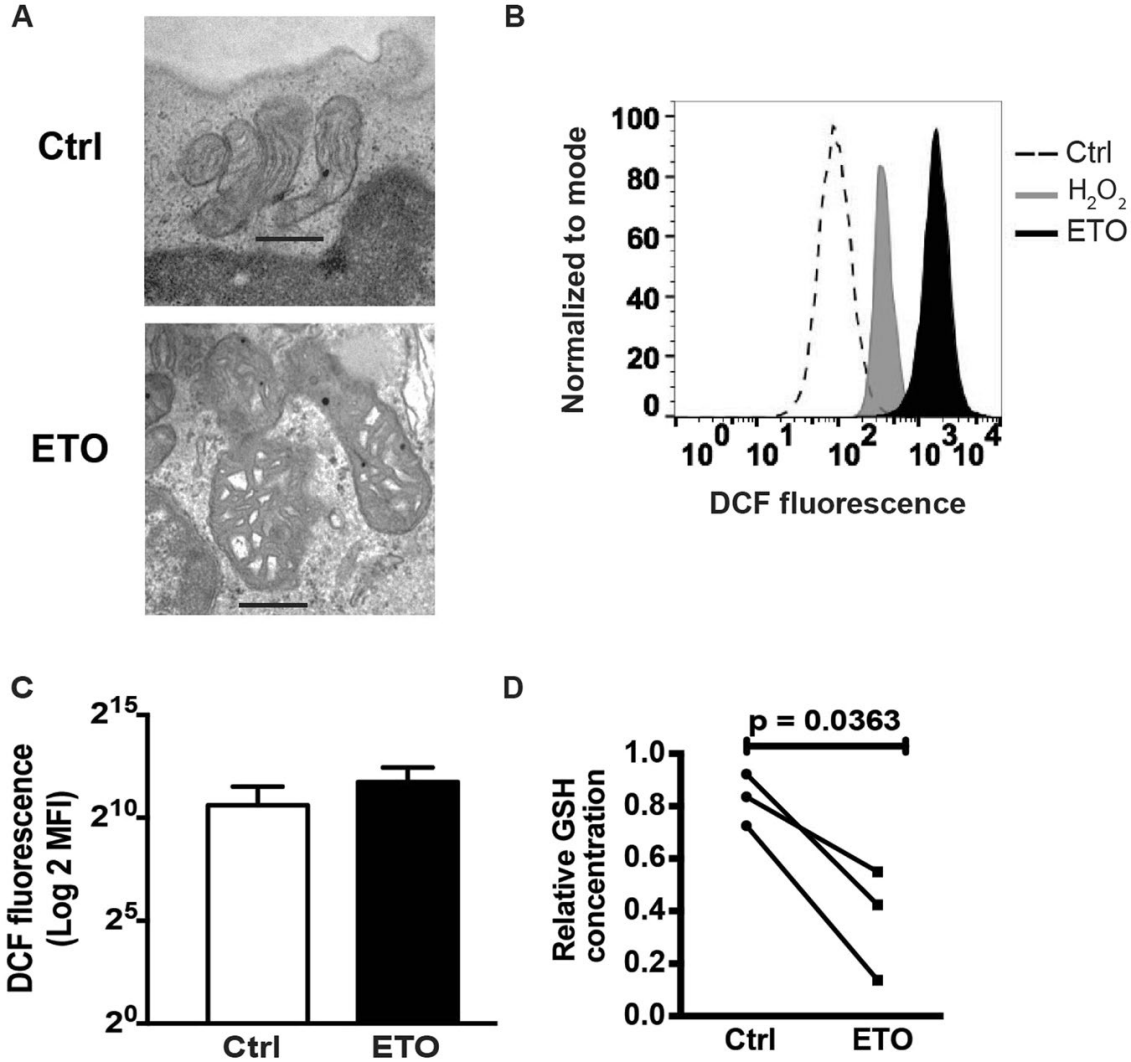
Commonly used concentrations of ETO induce severe oxidative stress in CD28-costimulated T cells. (**A**) Activated T cells were expanded in the presence of 50 μM until restdown. After thawing, these cells were processed for transmission electron microscopy. Representative images from two independent experiments are shown. Scale bars represent 500 nM. (**B**) ROS production in T cells treated with ETO for 4 hrs was measured by 2′,7′ dichlorofluorescein (*DCF*) fluorescence. Shown are the representative flow cytometry histograms (n = 5) of samples treated with either 50 μM H_2_O_2_ or 50 μM ETO. (**C**) Mean fluorescence intensities of cells treated with either control or ETO. (**D**) GSH levels of activated T cells treated with either control or 50 μM ETO over 5 days were quantified by LC-MS. Levels of reduced glutathione (Light) were assessed after normalizing to an isotopically-labeled standard (Heavy). The mean GSH levels ± SD were determined from triplicate measurements per condition in each experiment. Paired data (Ctrl vs ETO) from three individual experiments with separate donors are shown. GSH levels were decreased in ETO-treated T cells (Paired t-test analyses; *p < 0.05).

Based upon the putative role of metabolites for mitochondrial health and the importance of mitochondria to T cell biology, we evaluated mitochondrial morphology in control and ETO-treated T cells by electron microscopy. We observed that mitochondria from T cells cultured with 50 μM ETO demonstrate significant matrix swelling (Fig. 4A), a morphologic change consistent with an opening of the mitochondrial permeability transition pore (mPTP). As the mPTP is well know to be regulated by reactive oxygen species (ROS)^20,21^ and represents a well know pathway for ROS and calcium release from mitochondria^22^, we postulated that the effects of ETO on mitochondria may be related to excessive ROS generation. Following 4 hours of treatment with 50 μM ETO, proliferating T cells show a marked increase in DCF fluorescence that exceeds the ROS production following hydrogen peroxide treatment (Fig. 4B,C). Supporting these results, we also observed a significant decrease in GSH concentrations relative to oxidized glutathione in ETO-treated cells relative to untreated T cells (Fig. 4C). These data indicate that T cells cultured in ETO at concentrations commonly used to inhibit LCFAO are subjected to severe oxidative stress.

## Discussion

By combining metabolic studies with genetic approaches to manipulate CPT1a, we have shown that the widely used CPT1a inhibitor, ETO exhibits significant effects unrelated to cpt1a inhibition at concentrations in excess of 5 μM. These off-target effects are associated with inhibition of oxidative metabolism and induction of severe oxidative stress in T cells. In addition to being highly toxic to cells by causing oxidative damage to DNA, lipids and proteins, ROS can induce many signals within cells including MAPK activation^23^ and calcium release from intracellular compartments^24,25^.

The off-target effects that we have observed with ETO illustrate the challenges of using inhibitors, and indicate that results derived from ETO treatment used for the purpose of CPT1A inhibition must be interpreted cautiously, especially at high concentrations. Recently, ETO at concentrations above 10 μM has been reported to inhibit the mitochondrial adenine nucleotide transporter as well as complex Ι of the electron transport chain^26^. It’s well established that the complex Ι inhibitor, Rotenone, impairs mitochondrial function by increasing ROS production^27,28^ suggesting that ETO and Rotenone induce ROS though similar mechanisms. The concentration threshold at which off-target effects appeared following ETO treatment^26^ are well supported by our studies in T cells.

Discrepancies between pharmacologic and genetic inhibition of CPT1a-mediated transport of LCFA have been previously described. Studies using ETO showed an indispensable role of FAO in macrophage M2 polarization^14^. However, Finkel and coworkers showed that CPT2−/− macrophages differentiated towards an M2 lineage despite their inability to oxidize long chain fatty acids^15^.

In T cells, an essential role for LCFAO in the differentiation of central memory T cells has been established through the use of ETO at concentrations of 200 μM. These studies led to elaborate models of futile substrate cycles in murine T cells, whereby glucose derivatives are channeled into anabolic pathways supporting fatty acid synthesis. In response to antigen and costimulation, metabolic programs emphasizing LCFAO may indeed sustain mitochondrial function^9^, support mitochondrial ultrastructure^29^, and be permissive for memory T cell differentiation^10^. However, our findings raise caution about interpreting data generated by the use of the pharmacologic inhibitor, ETO especially when used at concentrations higher than 10 μM as many of the phenotypes might be a consequence of oxidative stress rather than CTP1a inhibition.

Our study may also help explain the observed hepatotoxicity of ETO in human clinical trials that has limited its development for manipulation of FA metabolism in the treatment of diabetes, inflammation and cancer. Although the mechanism of liver injury was not evaluated in the phase II study evaluating ETO in chronic heart failure^30^, oxidative stress is a well-known mechanism for liver injury^31^. The induction of oxidative stress with mitochondrial pathology comparable to that observed in T cells in our studies lend support to this mechanism of toxicity for ETO *in vivo*^32^.

## Materials and Methods

### Cell Culture

Primary human leukocytes (PBLs) from healthy male and female volunteers, averaging 34 years of age, were collected at the University of Pennsylvania’s Apheresis Unit. Informed consent was obtained from all participants prior to collection. All methods and experimental procedures were approved by the University of Pennsylvania Institutional Review Board. T cells were purified at the University’s Human Immunology Core by negative selection using the RosetteSep T cell enrichment Cocktail. Following isolation, T cells were cultured in growth medium (GM) comprising RPMI 1640 (Lonza) supplemented with 10% FBS (Hyclone), 10 mM HEPES, 2mM L-glutamine 100 U/ml penicillin G and 100 µg/ml streptomycin. For T cell activation, 4.5 µm Dynabeads containing immobilized anti-human CD3 and anti-human CD28 (Life Technologies) were used at a ratio of 3 beads to 1 cell. T cells were maintained in culture at a concentration of 0.8–1.0 × 10^6^ cells/ml through regular counting by flow cytometry using CountBright beads (BD Biosciences), a viability marker (Viaprobe) and mAbs to either human CD4 or CD8 as described in^33^. Where indicated, activated T cells (day3) were treated with 50 μM ETO throughout expansion. Lymphocytes were cultured at 37 °C, 20% O_2_ and 95% humidity with 5% CO_2_ unless otherwise stated.

### Isotope labeling

For glucose-labeled isotope experiments, cells were cultured in RPMI 1640 w/o D-glucose, w/o L-glutamine (Biological Industries) supplemented with 10% dialyzed FBS (Life Technologies), 2mM L-glutamine (Life technologies), and 10 mM ^13^C_6_ glucose (Sigma-Aldrich) for 1 hour. For glutamine-labeled isotope experiments, cells were cultured in RPMI 1640 w/o D-glucose, w/o L-glutae, supplemented with 10% dialyzed FBS, 10 mM D-glucose (Sigma-Aldrich), and 2 mM ^13^C_5_ glutamine (Sigma-Aldrich) for 1 hour. In each experiment, all measurements were performed in triplicate. Three independent experiments with separate donors were analyzed.

### Short-chain Acyl-CoA Extraction

Extractions were performed as described previously^34^. Briefly, lymphocytes were centrifuged at 1200 rcf for 5 min. Cell pellets were resuspended in 750 µl of ice-cold 10% trichloroacetic acid and pulse-sonicated using a sonic dismembrator (Fisher). The samples were centrifuged at 15,000 rcf for 15 min and the supernatants were purified by solid phase extraction. Briefly, Oasis HLB 1-ml (30 mg) solid-phase extraction columns were conditioned with 1 ml methanol, followed by 1 ml of H_2_O. The supernatants were applied to the column and washed with 1 ml of H_2_O. The analytes were eluted in methanol containing 25 mM ammonium acetate. The eluates were dried under N_2_ gas and resuspended in 50 µl of 5% 5-sulfosalicylic acid. Samples were analyzed by LC/ESI/MS/MS using an Ultimate 3000 Quaternary UHPLC coupled to a Q Exactive Plus mass spectrometer (Thermo Fisher Scientific) operating in the positive ion mode as previously described^35^.

### Metabolite Quantification

Primary human T cells were activated with Dynabeads and expanded for 7 days. T cells were maintained in culture at a concentration of 0.8–1.0 × 10^6^ cells/ml through regular counting. Where indicated, activated T cells (day3) were treated with 50μM ETO throughout expansion. The cells were harvested in 1 ml of ice-cold 10% w/v trichloroacetic acid solution, and every sample was spiked with an equal amount of ^15^N_1_^13^C_3_-labeled acyl-CoA internal standard (generated as described previously (Snyder *et al*., 2015)). Acyl CoA’s were extracted as described above. Absolute metabolite levels were determined by comparison with standard curves generated by serial dilution of acyl-CoA standards (Sigma-Aldrich) dissolved in trichloroacetic acid at known concentrations. Standards were spiked with internal standard and extracted in the same manner as the cell samples.

### Mitochondrial Respiratory Features as a function of glutamine concentration

Mitochondrial function was assessed using an extracellular flux analyzer (Agilent/Seahorse Bioscience). Individual wells of an XF96 cell culture microplate were coated with CellTak in accordance with the manufacturer’s instructions. The matrix was adsorped overnight at 37 °C, aspirated, air dried, and stored at 4 °C until use. Bulk T cells were activated with Dynabeads and expanded for seven days. To assay mitochondrial function in response to varying glutamine levels, T cells were centrifuged at 1200 g for 5 min. Cell pellets were re-suspended in XF assay medium (non-buffered RPMI 1640) containing 5.5 mM glucose, varying concentration (0, 2, and 8 mM) of L-glutamine and 1 mM sodium pyruvate. T cells were seeded at 0.3 × 10^6^ cells/well. The microplate was centrifuged at 1,000 g for 5 min and incubated in standard culture conditions for 60 min. During instrument calibration (30 min), the cells were switched to a CO_2_-free, 37 °C, incubator. XF96 assay cartridges were calibrated in accordance with the manufacturer’s instructions. Cellular oxygen consumption rates (OCR) were measured under basal conditions and following treatment with 1.5 μM oligomycin A, 1.5 μM fluoro-carbonyl cyanide phenlhdrazone (FCCP) and 50 μM etomoxir (ETO).

### Mitochondrial Respiratory Features as a function of ETO treatment

T cells expressing either scramble or CPT1A shRNA were seeded on Cell-Tak coated plates at 0.3e6 cells/well and the mitochondrial properties were assessed by Seahorse Assay as described above. Assay medium consisted of non-buffered RPMI 1640 containing 10 mM glucose, 2 mM of L-glutamine, and 5 mM hepes. Cellular oxygen consumption rates (OCR) and ECAR levels were measured under basal conditions and following treatment with either 5 μM or 50 μM ETO. In separate experiments, untransduced T cells were cultured in assay medium that was supplemented with 1 mM sodium acetate prior to the introduction of either 5 μM or 50 μM ETO through Port A of the instrument.

### Abs and flow cytmetry

To assess differentiation status, cells were stained at the indicated timepoints with a panel of mabs to CD4, CD8, CD45RO, CCR7, and LIVE/DEAD Aqua dye (Invitrogen). In parallel assays, FMO gating controls were performed.

Flow cytometry was performed using an LSR Fortessa (BD Biosciences, San Jose, CA), and data were analyzed using Flowjo software (Treestar, Ashland, OR).

### ROS assays

T cells were stimulated with Dynabeads and logarithmically expanded over several days. Cells were then resuspended in phenol-free growth medium and treated with 20 μM dichlorofluorescein diacetate (DCFH-DA) for 30 min under standard culture conditions. Where indicated, either 50 μM etomoxir or 50 μM H_2_O_2_ were added to select wells. After 4–5 hrs, reactive oxygen species formation as measured by the abundance of the fluorescent product 2′, 7′-DCF was determined by flow cytometry. Five independent experiments with separate donors were analyzed.

### shRNA-GFP lentiviral plasmid construction and infection

The puromycin resistance gene in 5 individual shRNA lentiviral plasmids against CPT1A (Open Biosystems) was replaced with GFP cDNA using standard molecular biology techniques. Briefly, a 1.4 kb GFP cDNA insert was subcloned from the lentiviral PELPS-GFP plasmid into KpnΙ and BamHΙ sites in the pLKO.1 shRNA plasmid. The efficacy of each individual lentiviral plasmid was determined by immunoblot analysis. Lentiviral infections were performed as described^36^. Lymphocytes expressing shRNA against CPT1A were compared with the corresponding control plasmid (Open Biosystems) that we also engineered to express GFP instead of puromycin resistance. The control plasmid encoded a scrambled shRNA sequence from the human β-actin gene. The efficiency of lentiviral infection ranged from 60–90% across experiments. In assessments of cell proliferation, enumeration was performed using bead-based counting methods following gating on GFP + cells. The titers for scramble-GFP and shRNA-CPT1A-GFP viral supernatants were 9.45e6 and 7.34e6 TU/ml, respectively.

### GSH Assays

Primary human T cells were activated with Dynabeads and expanded for 7 days. T cells were maintained in culture at a concentration of 0.8–1.0 × 10^6^ cells/ml through regular counting. Where indicated, activated T cells (day3 onwards) were treated with 50 μM ETO. On day 6, control vs ETO-treated T cells were prepared in triplicate and cultured overnight. The following morning these cells were washed three times with PBS before being harvested in 1 mL ice-cold methanol/water (4/1 v/v). Each sample was spiked with 10 μg [^13^C_2_, ^15^N_1_]-glutathione heavy internal standard (Cambridge Isotope Laboratories, MA, USA). The samples were transferred to 1.6 mL Eppendorf tubes, in which they were pulse-sonicated for 30 s with a probe tip sonicator and centrifuged at 16,000 g for 10 min. The supernatant was transferred to a new tube and dried under nitrogen. The residue was reconstituted in 50 μL 5% sulfosalicyclic acid.

An UltiMate 3000 UHPLC system equipped with a refrigerated autosampler (6 °C) and a column heater was used. Gradient and column conditions were performed as described in^37^. MS analysis was conducted on a Q Exactive^TM^ HF Hybride Quafrupole-Orbitrap^TM^ mass spectrometer (Thermo Scientific, San Jose, CA, USA) equipped with a HESI II source operating in positive mode. The operating conditions were as following: spray voltage 3500 V; capillary temperature 325 °C; auxiliary gas heater temperature 125 °C; S-lens 55. The sheath gas (nitrogen) and auxiliary gas (nitrogen pressures were 30 and 10 (arbitrary units), respectively. In-source CID was 1.0 eV, resolution was set as 30,000. Levels of GSH were based on exact mass analyzed by Xcalibur software (version 2.6). Alternatively, samples were analyzed by an Agilent 1200 series HPLC system coupled to an Agilent 6460 triple quadrupole mass spectrometer using conditions as described in^37^. The ion transitions of GSH were described as before^38^. Relative GSH levels were determined by comparing the light (endogenous GSH) versus heavy (internal standard) glutathione ratio. Three independent experiments with separate donors were analyzed.

### Transmission Electron Microscopy

T cells were logarithmically expanded in the presence of either vehicle or 50uM ETO for several days and subsequently frozen at restdown. Upon thaw, the cells were prepared with Penn’s Electron Microscopy Resource Laboratory and imaged with the Jeol-1010 microscope.

### Immunoblotting

CPT1A protein expression was assessed 5 days following shRNA lentiviral infection. Cells were lysed in RIPA-2 (50 mM Tris-HCl, pH8.0, 150 mM NaCl, 1% NP-40, 0.5% deoxycholic acid, 0.1% SDS) containing protease (Mini Complete; Roche) and phosphatase inhibitors (PhosSTOP; Roche). Equal amounts of lysate were separated by SDS-PAGE and transferred electrophoretically to a PVDF membrane (Millipore). Membranes were incubated in 5% nonfat milk in TBS (20 mM Tris, 135 mM NaCl) containing 0.1% Tween-20 (TBS-T) for 1 hr. After blocking, the membranes were probed with a 1:500 dilution of anti-CPT1A (Cell Signaling) in 0.5% nonfat milk in TBS-T. After a series of washes in TBS-T, the membranes were incubated with followed by a 1:10,000 dilution of HRP-conjugated goat anti-rabbit IgG (Cell Signaling). Antibody binding was detected using West Femto SuperSignal chemiluminescent reagents (ThermoScientific). Relative protein loading was determined with a 1:2,000 dilution of mouse monoclonal antibody to β-Actin (Cell Signaling) followed by a 1:5,000 dilution of HRP-conjugated sheep anti-mouse IgG (Amersham).

### Statistical Analysis

Student’s *t* test for paired data, or a two-way ANOVA using the Bonferroni method were performed using GraphPad Prism version 4.0a (GraphPad Software). A p value of 0.05 was considered statistically significant.

## Electronic supplementary material

Supplementary Information
