# Supplementary material for: The CPT1a inhibitor, etomoxir induces severe oxidative stress at commonly used concentrations
Source: Sci Rep. 2018 Apr 19;8:6289. doi: 10.1038/s41598-018-24676-6 (PMC5908836; doi:10.1038/s41598-018-24676-6)
Supplement: Supplementary file 1 — Supplementary Information [file 41598_2018_24676_MOESM1_ESM.pdf]

Supplementary Information

The CPT1a inhibitor, etomoxir induces severe oxidative stress at commonly used concentrations

Authors: *Roddy S. O'Connor[1, 4], Lili Guo[2], Saba Ghassemi[1, 4], Nathaniel W. Snyder[3], Andrew J. Worth[2], Liwei Weng[2], Yoonseok Kam[5], Benjamin Philipson[6], Sophie Trefely[3], Selene Nunez-Cruz[1, 4], Ian A. Blair[2], Carl H. June[1, 4], Michael C. Milone[1, 4]*

[1]Center for Cellular Immunotherapies, Perelman School of Medicine at the University of Pennsylvania, Philadelphia, PA. [2]Penn SRP center, Center of Excellence in Environmental Toxicology, and Department of Systems Pharmacology and Translational Therapeutics at the University of Pennsylvania, Philadelphia, PA. [3]A.J. Drexel Autism Institute, Drexel University, Philadelphia, PA. [4]Department of Pathology and Laboratory Medicine, Perelman School of Medicine at the University of Pennsylvania, Philadelphia, PA. [5]Agilent Technologies Inc, Lexington, MA.[6]University of Pennsylvania School of Medicine, Philadelphia, PA.

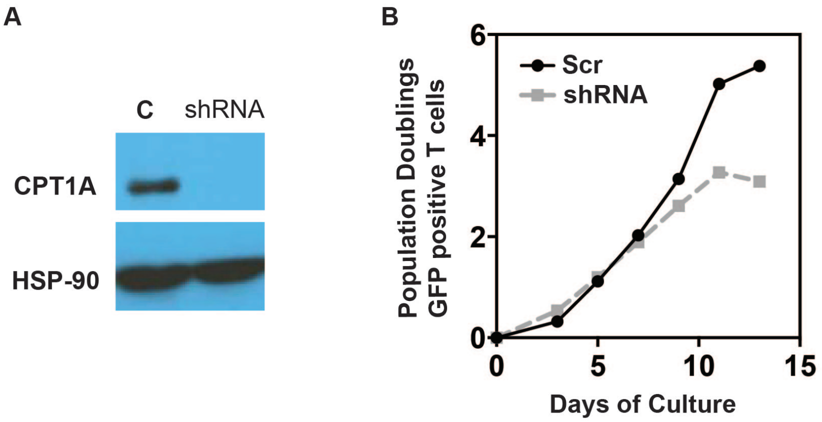

Supplementary Figure S1. CPT1A knockdown impairs T cell proliferation/survival

(A) After overnight stimulation with dynabeads, primary human T cells were infected with either shRNA against CPT1A or a control shRNA. These cells were expanded for 5 days and CPT1A expression was measured by immunoblot analysis. Relative protein loading was determined by immunoblotting for HSP-90. Representative data from 3 independent experiments are shown. (B)T cells were activated with dynabeads and infected with a bicistronic plasmid encoding a GFP transgene as well as shRNA against CPT1A. Cells were enumerated using flow cytometric, bead-based approaches described in the materials and methods. The number of population doublings is plotted as a function of time in the GFP+ subsets. Values are means ± S.E.M. from 4 independent experiments. (*P<0.05 for control vs shRNA CPT1A).

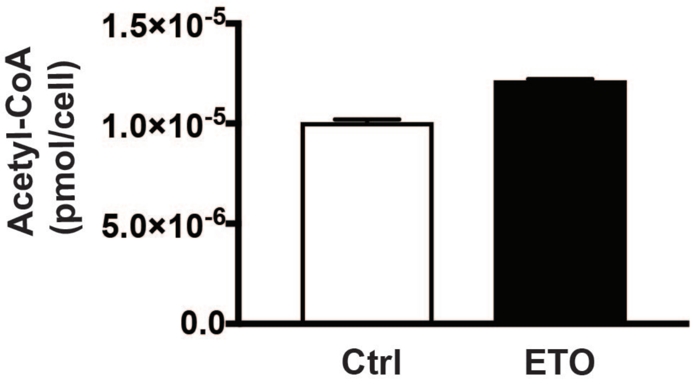

Supplementary Figure S2. Total cellular acetyl-CoA levels are unaffected by 50μM ETO.

Primary human T cells were activated dynabeads and expanded for 7 days. Where indicated, activated T cells (day3) were treated with 50μM ETO throughout expansion. Cells were seeded in triplicate and harvested in 10% TCA solution. Total cellular acetyl-CoA concentrations were determined by LC-MS. Values are means ± S.E.M. Representative data from two independent experiments with separate donors are shown.
